# An Apparent Trade-Off between Direct and Signal-Based Induced Indirect Defence against Herbivores in Willow Trees

**DOI:** 10.1371/journal.pone.0051505

**Published:** 2012-12-12

**Authors:** Kinuyo Yoneya, Masayoshi Uefune, Junji Takabayashi

**Affiliations:** Center for Ecological Research, Kyoto University, Otsu, Shiga, Japan; Trinity College Dublin, Ireland

## Abstract

Signal-based induced indirect defence refers to herbivore-induced production of plant volatiles that attract carnivorous natural enemies of herbivores. Relationships between direct and indirect defence strategies were studied using tritrophic systems consisting of six sympatric willow species, willow leaf beetles (*Plagiodera versicolora*), and their natural predators, ladybeetles (*Aiolocaria hexaspilota*). Relative preferences of ladybeetles for prey-infested willow plant volatiles, indicating levels of signal-based induced indirect defence, were positively correlated with the vulnerability of willow species to leaf beetles, assigned as relative levels of direct defence. This correlation suggested a possible trade-off among the species, in terms of resource limitation between direct defence and signal-based induced indirect defence. However, analyses of volatiles from infested and uninfested plants showed that the specificity of infested volatile blends (an important factor determining the costs of signal-based induced indirect defence) did not affect the attractiveness of infested plant volatiles. Thus, the suggested trade-off in resource limitation was unlikely. Rather, principal coordinates analysis showed that this ‘apparent trade-off’ between direct and signal-based induced indirect defence was partially explained by differential preferences of ladybeetles to infested plant volatiles of the six willow species. We also showed that relative preferences of ladybeetles for prey-infested willow plant volatiles were positively correlated with oviposition preferences of leaf beetles and with the distributions of leaf beetles in the field. These correlations suggest that ladybeetles use the specificity of infested willow plant volatiles to find suitable prey patches.

## Introduction

Besides the well-known direct defence mechanisms of plants against herbivores, many plants defend themselves indirectly by increasing the effectiveness of carnivorous natural enemies of these herbivores [1], [2]. For example, plants may constitutively provide a reward, such as food and/or shelter, to the carnivore species (e.g., [3], [4], [5], [6]). In addition, it has been widely observed that plants recruit specific carnivorous natural enemies of herbivores by emitting so-called “herbivore-induced plant volatiles” [7], [8], [9], [10], [11]. Here, we have termed this type of defence ‘signal-based induced indirect defence’. It is important to note that, in this kind of defence, the plants themselves do not produce a specific reward for the carnivores. Rather, it is the herbivores on a plant that are the rewards to the attracted carnivores. In such a reward system, the predators are assumed to prefer the volatile signal from plants infested by a sufficient number of suitable herbivores than that of non-infested plants (e.g., [12], [13]). Thus, important factors for the carnivores taking part in a signal-based induced indirect defence response are whether the number and species of herbivores on the plant constitute a sufficient reward. The goal of direct defence is to minimize the number of herbivores, while signal-based indirect defence aims to maintain a sufficient number of herbivores to reward the carnivores. Thus, it is evident that there is a conflict between direct defence and signal-based induced indirect defence in terms of the number of herbivores.

Given the contradictory requirements of direct and signal-based induced indirect defence, how these two defence systems could coexist in plants is unclear. In general, trade-offs among defence traits in plants have been predicted to result from resource limitation [14], [15], [16], because co-expression of multiple defences is thought to be costly for plants [17], [18]. The costs to plants of producing herbivore-induced carnivore attractants have been estimated to be low [19], [20]. However, Dicke and Sabelis [20] predicted that plants would be economize on energy spent producing carnivore attractants (i.e. signal-based induced indirect defence), even though the defence system consumed little energy. If so, resource limitation should lead to trade-offs between direct defence and signal-based induced indirect; plant species with lower levels of direct defence against a herbivore should invest more in signal-based induced indirect defence than those with higher levels of direct defence.

The leaf beetle, *Plagiodera versicolora*, is a specialist herbivore that feeds on leaves of Salicaceae. The predatory ladybeetle, *Aiolocaria hexaspilota*, is a specialist predator of *P. versicolora* larvae. *Aiolocaria hexaspilota* is known to be attracted to volatiles from a willow species (*Salix eriocarpa*) infested by *P. versicolora* larvae [21]. Furthermore, *A. hexaspilota* is preferentially attracted to volatiles from uninfested *S. eriocarpa* over clean air plus prey in a Y-tube olfactometer (Data S1). In the field, *P. versicolora* uses several sympatric *Salix* species as hosts. Each of these species is expected to exhibit signal-based indirect defence (either induced or constitutive) against *P. versicolora* by attracting its natural enemies, including *A. hexaspilota.* The relative vulnerability of different willow species to leaf beetles differs under field conditions. It is likely that ladybeetles locate their prey in the field by detecting various blends of volatiles from different willow plant species infested by the same leaf beetles.

To clarify the relative significance of direct and indirect defences in plants, we focused on tritrophic systems consisting of seven willow species that were indigenous to Japan, leaf beetles (*P. versicolora*), and ladybeetles (*A. hexaspilota*) occurring under natural conditions in Japan. A series of laboratory experiments were conducted to determine: (1) the relative preferences of ladybeetles to volatiles offered simultaneously from six willow species; (2) the vulnerability of six willow plant species to leaf beetle larvae; (3) the oviposition preferences of leaf beetle adults for the six willow plant species; (4) the distributions of leaf beetles in the field; and (5) the degree of chemical specificity of leaf volatiles emitted from leaves of the six willow species (either uninfested or infested by leaf beetle larvae). We first detected relative levels of signal-based indirect defence (either constitutive or induced). Subsequently, the relationship between direct defence and signal-based induced indirect defence mechanisms was assessed based on correlations between (1) and (2), (3), (4), and (5) and principle coordinates analyses (PCoA) of the chemical composition of the leaf volatiles emitted by the six infested willow species.

## Materials and Methods

### Insects

Adults and egg clutches of the willow leaf beetle, *P. versicolora*, were collected from the floodplain of the Yasu River (43°N, 141°E) in Shiga Prefecture, Japan, between April and October 2006. All investigations and collections of plants and insects were approved by the Ministry of Land, Infrastructure, Transport and Tourism of Kinki Regional Development Bureau Biwako Office, which manages the field area. Protected species were not sampled. Colonies were maintained on willow leaves in a climate-controlled chamber (25±3°C, 50−70% RH, light-dark cycle 18∶6 h), as described in our previous study [21]. Clutches of ladybeetle *A. hexaspilota* eggs were collected in the field between May and July 2006. Colonies were maintained in a similar climate controlled chamber, as described previously [21].

### Plants

During May 2006, a total of 50 1–2-year-old shoots (18 cm long; no leaves at that time of year) were cut from seven species of willow tree (*S. eriocarpa*, *Salix integra*, *Salix gracilistyla*, *Salix triandra*, *Salix chaenomeloides*, *Salix jessoensis*, and *Salix miyabeana*) growing in the floodplain of the Yasu River in Shiga Prefecture, Japan. The shoots were maintained with their basal sections in water for about 10 days until root emergence. They were then individually potted in sand (9 cm diameter×7.5 cm high pots), and the substrate was supplied with fertilizer (Hyponex, HYPONeX Japan, Osaka, Japan) every 2 weeks. The potted shoots were maintained in a greenhouse (25±3°C, light-dark cycle 18∶6 h) for 1 month until the newly emerged shoots were approximately 15–20 cm high with about 15 leaves. These shoots, which are the type predominantly fed on by leaf beetles under field conditions, were challenged by leaf beetles in the experiments.

Potted plants of each of the seven *Salix* species were used as the odour sources in each experiment. To prepare plants for herbivore infestation, 10±5 early second instar larvae of *P. versicolora* were introduced to shoots to ultimately produce a total of ∼2 cm^2^ of damaged area on three to four leaves per shoot (*S. miyabeana* was infested with five larvae; *S. eriocarpa, S. jessoensis*, and *S. chaenomeloides* with 10 larvae; and *S. integra*, *S. gracilistyla*, and *S. triandra* with 15 larvae). Larvae were randomly chosen from the culture, transferred to sixth leaf from the top of an undamaged shoot and allowed to feed for 1 day. Prior to the experiment, the leaf beetles and their faeces were removed from the plant with a piece of moist paper towel to exclude the effects of odour from leaf beetles and faeces on the behaviour of the ladybeetles.

### Relative Preferences of Ladybeetles to Volatiles from Willow Plants

We previously reported that *A. hexaspilota* have a robust ability to discriminate volatiles; the ladybeetle adults showed no preference for *S. eriocarpa* infested by leaf beetle adults (non-prey) over intact plants, but were more attracted to willow plants infested by leaf beetle larvae (prey) than to intact plants (21). Chemical analyses showed that the volatiles emitted by adult-infested and t larvae-infested leaves were different in quantity, but not in quality, of six compounds (21), further suggesting the robustness of the ladybeetle response to infested-leaf volatiles. Thus, as an index of the signal-based induced indirect defence level, the relative preference of ladybeetles for volatiles from willow plants with either infested or uninfested leaves was observed using an eight-choice olfactometer (Figure S1) under laboratory conditions (25±2°C and 55±5% RH). Each division of the olfactometer was connected to a polyethylene bag (2 L) containing an odour source (the shoot of a potted infested plant, a potted uninfested plant, or clean air). Clean air and the seven willow plant species were randomly positioned in the olfactometer in each bioassay. Air was cleaned using activated charcoal in a bottle (500 mL) before being sent to each odour source (1.5 L/min for each arm). The eight-choice olfactometer was set up in a draft chamber to allow a vertical flow of air from the bottom to the top. Ladybeetle adults were individually introduced at a starting point on a plate (9 cm diameter, 7.5 cm high) that was positioned at the centre of the eight volatile areas. The residence time of a ladybeetle in each area over a period of 10 min was measured. Ladybeetles that did not walk within 3 min were interpreted as having made “no choice”, and were excluded from statistical analyses. Each experiment was conducted over 3 to 4 experimental days. Treated plants were used once for each replicate, and 4–7 ladybeetles were used in each replicate. In total, 30 ladybeetles were used for this experiment. Individual ladybeetles were used only once. The olfactometer was washed after each experimental run.

### Vulnerability of Seven Willow Plant Species to Leaf Beetle Larvae

As a relative index of direct defence levels, the leaf area damaged by leaf beetles was measured for each of the seven willow species under laboratory conditions (25±2°C and 55±5% RH). A potted plant ∼20 cm long with newly emerged shoots was placed in a plastic cage (21×19×33 cm). Five leaf beetle larvae hatched from the same clutch of eggs were placed on one of the leaves. The area of the plant damaged by the leaf beetles before pupation was measured using an area meter (model CI-202, CID Inc., Camas, WA, USA). These experiments were repeated ten times. The vulnerability of each willow species to leaf beetle larvae would be due not only to resource availability to larvae, but also to other factors such as apparency to larvae, nutritional value, and other factors. In this study, the definition of direct defence involved such possible factors affecting willow resistance to herbivores.

### Oviposition Preferences of Leaf Beetles for the Seven Willow Species

Potted plants of the seven willow species were placed into an acrylic cage. Seven pots were arranged heptagonally (70 cm in diameter). The positions of the seven plants were changed randomly to eliminate positional preferences by the beetles. The cage had two mesh windows (70 cm^2^) on the two lateral sides. When willow plants are infested by leaf beetles in nature, a one-year-old shoot of roughly the same size used in this study hosts ca. three leaf beetle females, so in this study ten *P. versicolora* females were released into the middle of the cage. The leaves on which eggs were laid were removed at 3-day intervals. The total number of eggs laid by leaf beetles on each potted plant over a period of a week was counted. This value was defined as an index of oviposition preference by leaf beetles. This experiment was replicated 13 times on different experimental days. In four replicates, the females did not lay eggs, probably due to age and/or nutritional factors. These replicates were excluded from further analyses.

### Distribution of Leaf Beetles in the Field

The distribution of ladybeetles in the floodplain of the Yasu River was recorded on 15 May 2007. Adults of *A. hexaspilota* started to lay eggs at the beginning of May. Adults and larvae of *A. hexaspilota* preyed on the eggs and larvae of *P. versicolora* in the field. The numbers of eggs and larvae of leaf beetles on 10 1-year-old shoots from 10 plants of each of the seven *Salix* species used in this study were counted.

### Statistical Analyses of Bioassays

Kendall’s rank correlation coefficient was used to assess whether the relative preference for plant volatiles in ladybeetles was positively correlated with three variables: leaf area damaged by leaf beetle larvae; oviposition preference of leaf beetles for the seven willow species; and distribution of herbivores in the field.

### Chemical Analysis of Plant Volatiles

Volatiles were collected from plants with uninfested leaves and with leaves infested by leaf beetle larvae in a climate-controlled room (25±2°C). Infested plants were prepared as described above. Beetle larvae faeces present on each of the infested plants were carefully removed prior to the chemical analyses. Thus, the compounds recorded in the headspace of infested plants were of plant origin. A hexane solution of tridecane (0.5 µg/µL) impregnated into a piece of filter paper (1 cm^2^) was used as an internal standard.

A willow plant and the standard were placed in a glass bottle (3 L) fitted with two nozzles. One nozzle was connected to an air cylinder and the other to a glass tube packed with adsorbent (Tenax TA 20/35, 100 mg; I.D., 3 mm; length, 160 mm, GL Science, Japan). Purified air from the cylinder was directed into the glass bottle, and volatile compounds from the headspace of the bottle were collected on the adsorbent for 1.5 h at a flow rate of 100 mL/min. For both treatments (infested and uninfested plants), we replicated the volatile collection four times with each willow species.

The collected volatile compounds were analysed using a gas chromatograph (GC) (Agilent 6890N, Agilent Technologies, Inc., Palo Alto, CA, USA) equipped with an HP-5MS capillary column (Agilent; length 30 m, ID 0.25 mm, film thickness 0.25 µm) coupled with an Agilent 5973N quadrupole mass selective detector (MS) (70 eV). The system was equipped with a thermal desorption cold-trap injector (TCT; model CP4010; Chrompack, The Netherlands). Headspace volatiles collected on the adsorbent were released by heating in the TCT at 220°C for 8 min with He gas flow (1 mL/min). The desorbed compounds were collected in the TCT unit (SIL5CB-coated fused silica capillary) at −130°C. Flash heating of the cold-trap unit induced a sharp injection of the compounds into the capillary column of the GC. The GC oven temperature was programmed to increase from 40°C (5-min hold) to 280°C at a rate of 15°C/min. The headspace volatiles were tentatively identified by comparing their mass spectra with those from the Wiley databases (Wiley7N and Wiley275) and the database of National Institute of Advanced Industrial Science and Technology (SDBS compounds and spectral search; http://riodb01.ibase.aist.go.jp/sdbs/cgi-bin/direct_frame_top.cgi). Retention times of volatiles were further compared with those of standard compounds (Wako Chemicals, Osaka, Japan, and IFF Chemical, Jacksonville, FL, USA). Compounds for which no standards were available were regarded as tentatively identified when more than 90% of spectra matched those of the databases. The ion intensity of each peak was normalized by dividing the ion intensity of the internal standard by the weight of fresh leaves. The normalized data are referred to as ‘peak areas’.

### Statistical Analyses of Chemical Data

The peak area of each compound in each willow species, as well as the total peak area of the blend, was tested for correlation with the relative preferences of ladybeetles to each plant volatile using Kendall’s rank correlation test.

The Bray-Curtis dissimilarities of blends of volatiles between uninfested and infested plants within a species, which indicated the degree of specificity of plant volatiles to infested versus uninfested plants, were calculated. Kendall’s rank correlation coefficient was used to measure whether the relative preference for plant volatiles in ladybeetles correlated with the degree of specificity of infested plant volatiles within that plant species. To clarify whether the blends of volatiles differed among willow species and which volatile compound(s) explained the species specificity of the blend, we conducted PCoA on the Bray-Curtis dissimilarities. The compositions of the peak areas (log-transformed ratio of each peak area to total peak area) of all compounds were compared among plant species with a permutational multivariate analysis of variance (PERMANOVA; no. of permutations performed = 9999; [22], [23], [24]) based on Bray-Curtis dissimilarities of the log-transformed relative peak area of the volatile compounds.

## Results

### Relative Preferences of Ladybeetles to Volatiles from Willow Plants

**Figure 1:**
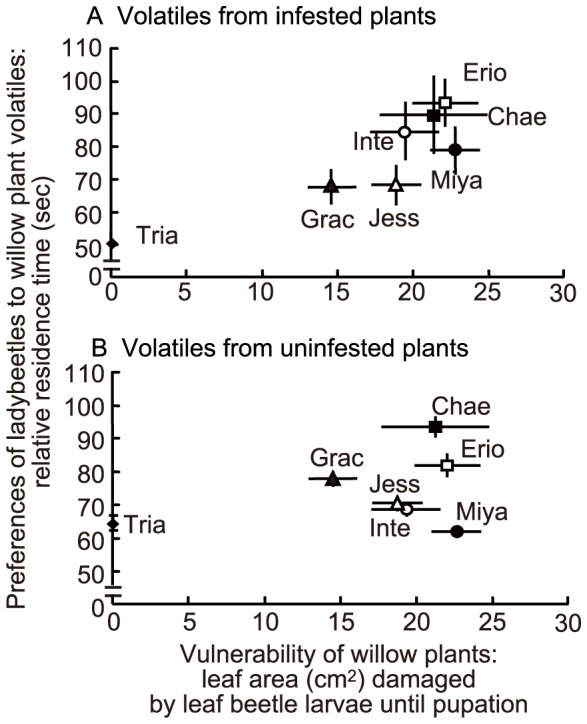
Relationship between vulnerability of willow plants and preferences of ladybeetles to willow plant volatiles. Leaf areas damaged by five larvae of leaf beetle *Plagiodera versicolora* until pupation was defined as an index of vulnerability of willow plants (mean ± S.E., N = 10). The relative residence time of predatory ladybeetles *Aiolocaria hexaspilota*, attracted by willow plant volatiles was defined as an index of the preferences of the ladybeetles (mean ± S.E., N = 30). Odour source: (A) infested plants and (B) uninfested plants. Erio: *Salix eriocarpa*; Chae: *S. chaenomeloides*; Inte: *S. integra*; Miya: *S. miyabeana*; Jess: *S. jessoensis*; Grac: *S. gracilistyla* and Tria: *S. triandra*.

**Figure 2:**
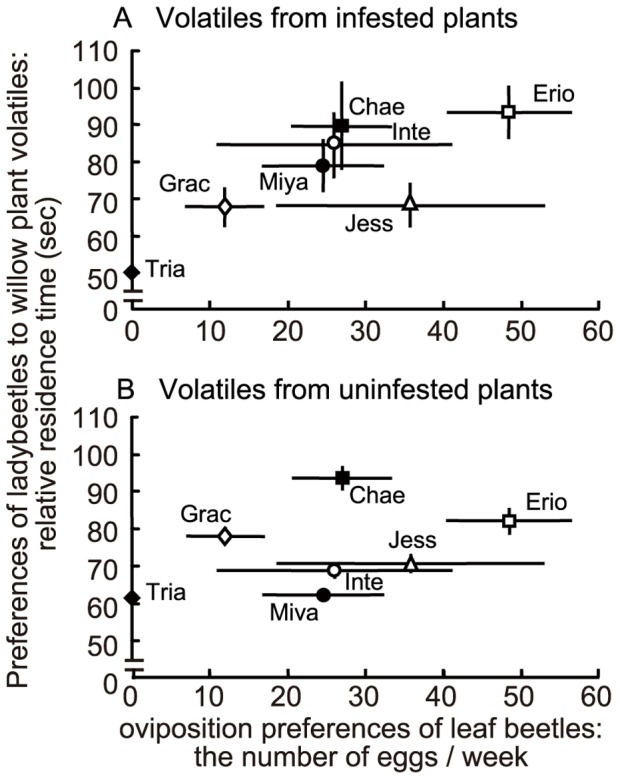
Relationship between oviposition preferences of leaf beetles and preferences of ladybeetles to willow plant volatiles. The number of eggs laid by leaf beetles *Plagiodera versicolora* was defined as an index of oviposition preferences of leaf beetles (mean ± S.E., N = 9). The relative residence time of predatory ladybeetles *Aiolocaria hexaspilota*, attracted by willow plant volatiles was defined as an index of the preferences of the ladybeetles (mean ± S.E., N = 30). Odour source: (A) infested plants and (B) uninfested plants. Erio: *S. eriocarpa*; Chae: *S. chaenomeloides*; Inte: *S. integra*; Miya: *S. miyabeana*; Jess: *S. jessoensis*; Grac: *S. gracilistyla* and Tria: *S. triandra*.

The relative preferences of ladybeetles for volatiles from leaves of uninfested plants of the seven willow species were, in descending order: *S. chaenomeloides* (Chae), *S. eriocarpa* (Erio), *S. gracilistyla* (Grac), *S. jessoensis* (Jess), *S. integra* (Inte), *S. triandra* (Tria), and *S. miyabeana* (Miya) (Y axis of Figure 1b). Preferences for leaf volatiles from infested willows were, in descending order: Erio, Chae, Inte, Miya, Jess, Grac and Tria (Y axis of Figures 1a and 2). Because *S. triandra* leaves were not infested by leaf beetle larvae under laboratory conditions, artificially damaged leaves were used as the odour source. Thus, data for *S. triandra* were excluded from statistical analyses in subsequent experiments. The relative preferences for infested-leaf and uninfested-leaf volatiles were considered to represent relative levels of signal-based induced indirect defence and signal-based constitutive indirect defence [25], [26], respectively.

### Relationships between Direct and Signal-based Indirect Defence in Six Species of Willow

The relative preference of ladybeetles for volatiles from leaves of infested willow species (Figure 1, Y axis) were positively correlated with vulnerability (leaf area damaged by leaf beetle larvae) (Kendall’s rank correlation coefficient = 0.60, *P*<0.05; Figure 1a). In contrast, the relative preference for volatiles from uninfested leaves (Figure 1b, Y axis) did not correlate with vulnerability (Kendall’s rank correlation coefficient = 0.20, *P*>0.05; Figure 1b).

The relative preference of ladybeetles for volatiles of leaves from infested plants of the willow species (Fig. 2a, Y axis) were positively correlated with oviposition preference of leaf beetle females for each willow species (Kendall’s rank correlation coefficient = 0.71, *P*<0.05; Fig. 2a). The relative preferences of predatory ladybeetles for volatiles from uninfested leaves of six willow species (Fig. 2b, Y axis) did not correlate with the oviposition preference of leaf beetle females (Kendall’s rank correlation coefficient = 0.43, *P*>0.05; Fig. 2b).

**Figure 3:**
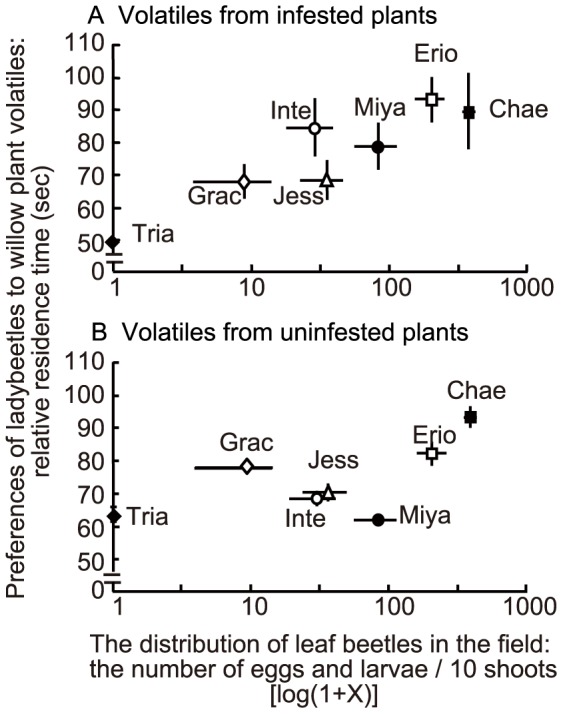
Relationship between distribution of leaf beetles and preferences of ladybeetles to willow plant volatiles. The distribution of leaf beetles *Plagiodera versicolora* was investigated by counting the numbers of eggs and larvae of the leaf beetles on shoots in the field (mean ± S.E., N = 10). The relative residence time of predatory ladybeetles *Aiolocaria hexaspilota*, attracted by willow plant volatiles was defined as an index of the preferences of the ladybeetles (mean ± S.E., N = 30). Odour source: (A) infested plants and (B) uninfested plants. Erio: *S. eriocarpa*; Chae: *S. chaenomeloides*; Inte: *S. integra*; Miya: *S. miyabeana*; Jess: *S. jessoensis*; Grac: *S. gracilistyla* and Tria: *S. triandra*.

The relative preference of ladybeetles for volatiles from leaves of infested willow species (Fig. 3, Y axis) positively correlated with the abundance of leaf beetles (eggs and larvae) in the field (Kendall’s rank correlation coefficient = 0.71, *P*<0.05; Fig. 3a). However, their relative preferences for volatiles from leaves of uninfested plants (Fig. 3b, Y axis) did not correlate with leaf beetle abundance (Kendall’s rank correlation coefficient = 0.33, *P*>0.05; Fig. 3b).

### Chemical Analyses

Seventeen volatile compounds were found to be emitted by the leaves of the six willow species that were infested by leaf beetle larvae (Table S1), whereas only seven different compounds were emitted by uninfested leaves of the seven willow species (Table S2).

**Figure 4:**
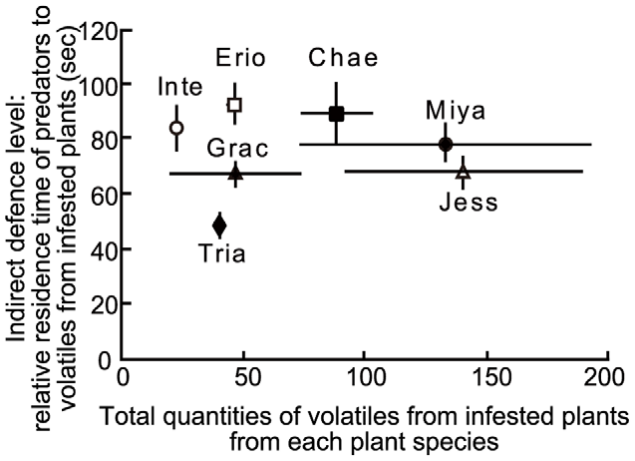
Total quantities of infested plant volatiles and indirect defence level. The relative residence time of predatory ladybeetles *Aiolocaria hexaspilota*, attracted by infested willow plant volatiles was defined as an index of induced indirect defence level of willow plant species (mean ± S.E., N = 30). Erio: *Salix eriocarpa*; Chae: *S. chaenomeloides*; Inte: *S. integra*; Miya: *S. miyabeana*; Jess: *S. jessoensis*; Grac: *S. gracilistyla* and Tria: *S. triandra*. The value of total quantities of infested plant volatiles represents mean (± S.E.) for four samples.

Neither the amount of each compound in each willow species (Kendall’s rank correlation test, *P*>0.05 for each compound; Table S1) nor the total amounts in each blend of volatiles was correlated with the relative preference of ladybeetles for volatiles from leaves of infested willow species (Kendall’s rank correlation test, *P*>0.05; Figure 4).

**Figure 5:**
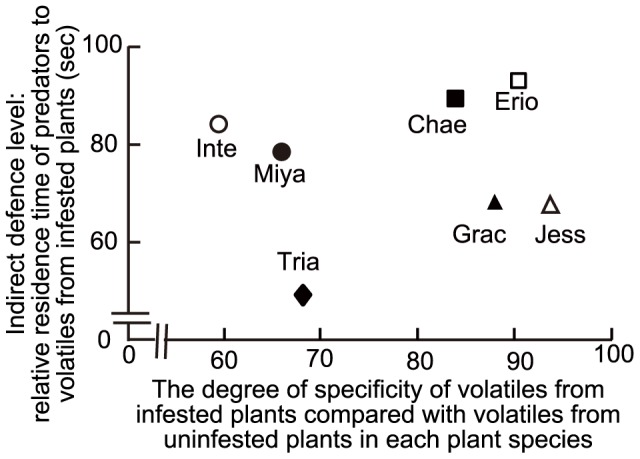
Degree of specificity of infested plant volatiles and preferences of ladybeetles to willow plant volatiles. The degree of specificity of infested-plant volatiles compared with uninfested-plant volatiles was indicated by the dissimilarity distances between volatiles from uninfested and infested plants within a plant species. The relative residence time of predatory ladybeetles *Aiolocaria hexaspilota*, attracted by willow plant volatiles was defined as an index of the preferences of the ladybeetles. Erio: *Salix eriocarpa*; Chae: *S. chaenomeloides*; Inte: *S. integra*; Miya: *S. miyabeana*; Jess: *S. jessoensis*; Grac: *S. gracilistyla* and Tria: *S. triandra*.

We then calculated the degree of specificity of infested-plant volatiles compared with uninfested-plant volatiles for each species. No significant correlation was found between the degree of specificity and the relative preferences of ladybeetles for volatiles from leaves of infested willow species (Kendall’s rank correlation coefficient = −0.2, *P = *0.57; Figure 5).

**Figure 6:**
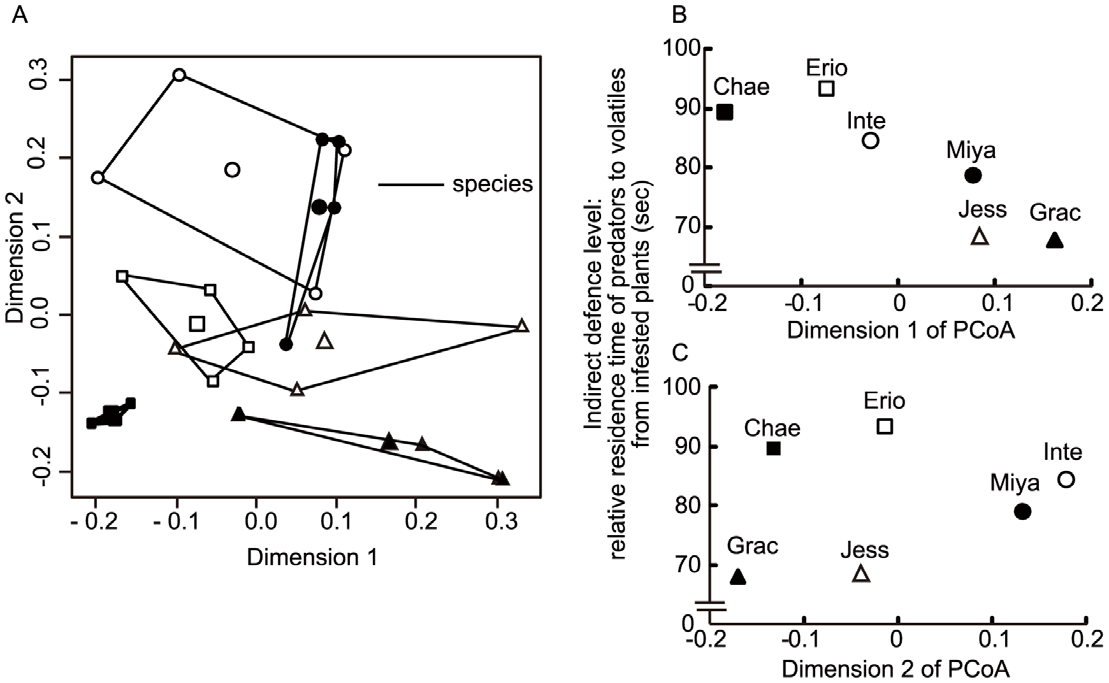
Principal coordinates analysis on volatiles composition of willow plant species. (A) Plots of axes 1 and 2 (shown in panels b and c) of the principal coordinates analysis of 28 samples based on volatiles composition (▪, *Salix chaenomeloides*; □, *S. eriocarpa*; ○, *Salix integra*; •, *S. miyabeana*; Δ, *S. jessoensis*; ▴, *S. gracilistyla*). The average score of each plant species is shown as a larger symbol. The first and second axes of the PCoA (Dim 1 and Dim 2) explained 30.4% and 27.6%, respectively, of the variation in association between samples, taking the absolute values of negative eigenvalues. (B and C) Relationships between the average scores of each plant species in the first (B) and second (C) axes of the PCoA and the relative residence times of predatory ladybeetles *Aiolocaria hexaspilota* attracted by infested plant volatiles. The scores (coordinates) of volatile compounds are shown in Table S3.

The first and second PCoA axes (Dimension 1 and Dimension 2 in Figure 6a) of 28 samples, based on the composition of volatile compounds from the six infested plant species, explained 30.4% and 27.6%, respectively, of the variation in the association between samples (based on the absolute values of the eigenvalues). Plant species had a significant impact on the blend of infested-plant volatiles (PERMANOVA, F = 4.8, P<0.001; Figure 6a). The relative preference of ladybeetles for volatiles from leaves of infested willow species (Figure 6b, Y axis) was positively correlated with the mean value of each species in Dimension 1 (X axis, Kendall’s rank correlation = −0.87, *P*<0.05; Figure 6b) but not in Dimension 2 (X axis, Kendall’s rank correlation = 0.33, *P*>0.05; Figure 6c).

## Discussion

The leaf areas damaged by leaf beetle larvae in six willow species, which were considered to reflect relative levels of direct defence, were positively correlated with the relative preference of ladybeetles for infested willow leaf volatiles, which were considered an indicator of relative levels of signal-based induced indirect defence. In contrast, these parameters were not correlated with the relative preferences of ladybeetles for uninfested willow leaf volatiles, which were considered to reflect relative levels of constitutive indirect defence. These data indicated that a willow species that is more vulnerable to willow leaf beetle larvae has higher signal-based induced indirect defence levels and vice versa, suggesting the possibility of trade-offs between direct defence and signal-based induced indirect defence. This finding raises the question of whether the suggested trade-offs are related to resource limitations. However, chemical analyses did not support this possibility, as shown below.

We have already reported that ladybeetles were attracted to the volatiles of *S. eriocarpa* infested by leaf beetles and to the compounds recorded in the headspace of infested *S. eriocarpa* plants [21]. The same compounds were recorded in the headspaces of the six species of infested willow plants examined here, but the relative composition of the volatiles differed. Thus, quantitative differences in volatile blends among the six infested willow species could affect the relative preference of ladybeetles to the infested-plant volatiles. However, no significant correlation was found between the levels of specificity of the infested leaf volatiles (i.e., an important factor determining the costs of production, if any, of a specific blend) and the relative preferences of ladybeetles (Figure 5). These results implied that the signal-based induced indirect defence levels in willow plants were not linked to the costs of production of species-specific induced volatiles. Taken together, the correlation between direct and signal-based induced indirect defence could not be explained by trade-offs in resource limitations. These correlations have been termed apparent trade-offs [27].

An intriguing proximate question is why such apparent trade-offs were observed in the interactions among six willow plants, willow leaf beetles, and ladybeetles. To answer this question, we investigated whether any major compounds were correlated with the preferences of predators using PCoA to extract the subset of volatile compounds that were representative of the interspecific variability in herbivore-induced volatile blends and comparing it with the relative preference of the predator. This is a reasonable method since carnivores are known to respond to different blends of herbivore-induced plant volatiles in different ways (e.g. [28], [21], [13]). (*E*)-*β*-ocimene, 2-methylbutanenitrile, and 3-methylbutanenitrile were representative of variability in the blends of volatiles from an infested plant, as indicated by high scores on the first PCoA axis for these compounds (Table S3). This axis was also correlated with ladybeetle preference (Figure 6b), suggesting that the mechanisms involved in the apparent trade-offs were associated with the preference of ladybeetles; i.e., ladybeetles evaluated differences in the volatile blends of the six infested willow plant species to select more suitable prey-infested plants. To determine whether this ladybeetle preference has a genetic base or whether learning is involved in the preference is a planned future study.

Next, we focused on ultimate factors involved in apparent trade-offs between direct and signal-based induced indirect defence. It is important to note the following two points. First, unlike compounds involved in direct defence, such as toxins and repellents, infested plant volatiles involved in a signal-based induced indirect defence do not result in the direct elimination of herbivores. Rather, the volatiles cause the influx of carnivores that act as bodyguards and that are capable of responding to infested-plant volatiles as signals indicating the presence of prey. In this situation, the quality of the reward for the predator in terms of prey suitability and quantity is crucial in determining the influx. Thus, the effectiveness of the signal-based induced indirect defence is predator context-dependent. Second, the attractiveness of infested plant volatiles is not an absolute factor but is determined by environmental conditions. For example, when a willow plant is standing next to a more vulnerable willow plant, ladybeetles might prefer the latter. Therefore, the costs and benefits to the plant in investing in signal-based induced indirect defence depend on what kinds of plants (or plant community) are growing nearby (plant community context-dependence). In this study, the relative preferences of ladybeetles for prey-infested willow plant volatiles were positively correlated with the oviposition preferences of leaf beetles and with the distributions of leaf beetles in the field. In contrast, these parameters were not correlated with the relative preferences of ladybeetles for uninfested willow leaf volatiles (Figures 2 and 3). Note that we used infested plants with similar levels of damage by leaf beetles to determine the potential preferences of ladybeetles for prey-infested willow plant volatiles. Thus, these data suggest that ladybeetles were able to respond to the relative specificity of volatiles from infested willow plants to find the most suitable prey patches (patches with more prey) among the six willow species. Furthermore, if one of the plant species shown in Figures 2 and 3 were removed, the relative preferences would be changed. Thus, the signal-based induced indirect defence of willow plants would be dependent on both the predator context and the plant community context, a situation which would cause apparent trade-offs.

In summary, the evolution of the production of carnivore attractants by an infested plant is related not only to the level of resources invested in producing volatile secondary chemicals attractive to carnivores, but also to the predator and plant-community contexts. As pointed out by Strauss and Irwin [29], the evolutionary dynamics of two interacting individuals belonging to the same or different species depend on the presence of other organisms in the community. We hypothesize that the apparent trade-offs between direct defence and signal-based induced indirect defence are based on the suitability of sympatric plant species for predators in term of rewards (quality and quantity of herbivores) in a local ecosystem. In this case, conspecific plants in communities with different tritrophic community structures might evolve differently with respect to signal-based induced indirect defence. To test this hypothesis, a comparison of direct and indirect defence traits among geographically different willow communities is needed.

## Supporting Information

Figure S1 Eight choice chamber. Volatiles from a shoot of a potted willow plant were directed to seven of eight air inlets; the eighth inlet received clean air. The positions of the eight odour sources were changed randomly to remove any positional preferences of the ladybeetles.
(TIF)

Table S1 Volatiles detected in the headspace of plants of the seven *Salix* species infested by *P. versicolora* larvae.
(DOC)

Table S2 Volatiles detected in the headspace of uninfested plants of the seven *Salix* species.
(DOC)

Table S3 Scores (coordinates) of volatile compounds. The scores were calculated by weighting the correlation between sample scores (coordinates: each plot in Figure 4a (PCoA)) and vectors of compounds by the variance explained by the first two PCoA axes scores obtained using the Bray-Curtis measurement.
(DOC)

Data S1 Preferential attraction of ladybeetles to volatiles from uninfested *Salix eriocarpa* over clean air plus prey.
(DOCX)

## References

[1] Karban R, Baldwin IT (1997) Induced responses to herbivory. Chicago: The University of Chicago Press.

[2] Schoonhoven LM, van Loon JJA, Decke M (2005) Insect-plant biology, 4th edn. London: Oxford University Press.

[3] Davidson DW, Mckey D (1993) Ant plant symbioses - stalking the chuyachaqui. Trends Ecol Evol 8, 326–332. 10.1016/0169-5347(93)90240-P 21236183

[4] Agrawal AA, Rutter MT (1998) Dynamic anti-herbivore defense in ant-plants: the role of induced responses. Oikos 83, 227–236.

[5] Heil M, Fiala B, Baumann B, Linsenmair KE (2000) Temporal, spatial and biotic variations in extrafloral nectar secretion by *Macaranga tanarius*. Funct Ecol 14,749–757.

[6] Ness JH (2003) *Catalpa bignonioides* alters extrafloral nectar production after herbivory and attracts ant bodyguards. Oecologia 134, 210–218. 10.1007/s00442-002-1110-6 12647162

[7] Takabayashi J, Dicke M (1996) Plant-carnivore mutualism through herbivore-induced carnivore attractants. Trends in Plant Science 1, 109–113.

[8] Dicke M, Vet LEM (1999) Between plants and predators In: Olff H, Brown VK, Drent RH editors. Herbivores. Oxford: Blackwell Science. pp. 483–520.

[9] Hilker M, Meiners T (2002) Induction of plant responses to oviposition and feeding by herbivorous arthropods: a comparison. Entomol Exp Appl 104, 181–192.

[10] Turlings TCJ, Gouinguené S, Degen T, Fritzsche-Hoballah ME (2002) The chemical ecology of plant-caterpillar-parasitoid interactions. In: Tscharntke T, Hawkins B editors. *Multitrophic Level Interactions*. Cambridge: Cambridge University Press. pp. 148–173.

[11] Arimura G, Matsui K, Takabayashi J (2009) Chemical and molecular ecology of herbivore-induced plant volatiles: proximate factors and their ultimate functions. Plant Cell Physiol 50, 911–923. 10.1093/pcp/pcp030 19246460

[12] Maeda T, Takabayashi J (2001) Production of herbivore-induced plant volatiles and their attractiveness to *Phytoseius persimilis* (Acari : Phytoseiidae) with changes of *Tetranychus urticae* (Acari : Tetranychidae) density on a plant. Appl Entomol Zool 36, 47–52.

[13] Shiojiri K, Ozawa R, Kugimiya S, Uefune M, van Wijk M, et al.. (2010) Herbivore-specific, density-dependent induction of plant volatiles: honest or “Cry Wolf” signals? PLoS ONE 5, e12161. 10.1371/journal.pone.0012161 PMC2923144 20808961

[14] Ballhorn DJ, Kautz S, Lion U, Heil M (2008) Trade-offs between direct and indirect defences of lima bean (*Phaseolus lunatus*). J Ecol 96, 971–980.

[15] Heil M, Bostock RM (2002) Induced systemic resistance (ISR) against pathogens in the context of induced plant defences. Ann Bot-London 89, 503–512. 10.1093/aob/mcf076 PMC4233886 12099523

[16] Koricheva J, Nykanen H, Gianoli E (2004) Meta-analysis of trade-offs among plant antiherbivore defenses: Are plants jacks-of-all-trades, masters of all? Am Nat 163, E64–E75. 10.1086/382601 15122510

[17] Mole S (1994) Trade-offs and constraints in plant-herbivore defense theory - a life-history perspective. Oikos 71, 3–12.

[18] Mauricio R (1998) Costs of resistance to natural enemies in field populations of the annual plant *Arabidopsis thaliana*. Am Nat 151, 20–28. 10.1086/286099 18811421

[19] Gershenzon J (1994) Metabolic costs of terpenoid accumulation in higher plants. J Chem Ecol 20, 1281–1328. 10.1007/BF02059810 24242341

[20] Dicke M, Sabelis MW (1992) Cost and benefits of chemical information conveyance. In: Roitberg BD, Isman MB editors. Insect chemical ecology. New York: Chapman & Hall. pp. 122–155.

[21] Yoneya K, Kugimiya S, Takabayashi J (2009) Can herbivore-induced plant volatiles inform predatory insect about the most suitable stage of its prey? Physiol Entomol 34, 379–386.

[22] Anderson MJ (2001) A new method for non-parametric multivariate analysis of variance. Austral Ecol 26, 32–46.

[23] Anderson MJ (2005) PERMANOVA: a FORTRAN computer program for permutational multivariate analysis of variance. Department of Statistics, University of Auckland, New Zealand.

[24] McArdle BH, Anderson MJ (2001) Fitting multivariate models to community data: a comment on distancebased redundancy analysis. Ecology 82, 290–297.

[25] Takabayash J, Dicke M, Posthumus MA (1991) Induction of indirect defense against spider mites in uninfested Lima bean leaves. Phytochemistry 30: 1459–1462.

[26] Horikoshi M, Takabayashi J, Yamaoka R, Yano S, Ohsaki N, et al (1997) *Cotesia glomerata* female wasps use fatty acids from plant-herbivore complex in host searching. Journal of Chemical Ecology 23: 1505–1515.

[27] van Ballegooijen WM, Boerlijst MC (2004) Emergent trade-offs and selection for outbreak frequency in spatial epidemics. PNAS 101, 18246–18250. 10.1073/pnas.0405682101 PMC539754 15604150

[28] De Moraes CM, Lewis WJ, Pare PW, Alborn HT, Tumlinson JH (1998) Herbivore-infested plants selectively attract parasitoids. Nature 393, 570–573.

[29] Strauss SY, Irwin RE (2004) Ecological and evolutionary consequences of multispecies plant-animal interactions. Annu Rev Ecol Evol Syst 35, 435–466.

