# Supplementary figures and images for: An Apparent Trade-Off between Direct and Signal-Based Induced Indirect Defence against Herbivores in Willow Trees
Source: PLoS One. 2012 Dec 12;7(12):e51505. doi: 10.1371/journal.pone.0051505 (PMC3520792; doi:10.1371/journal.pone.0051505)

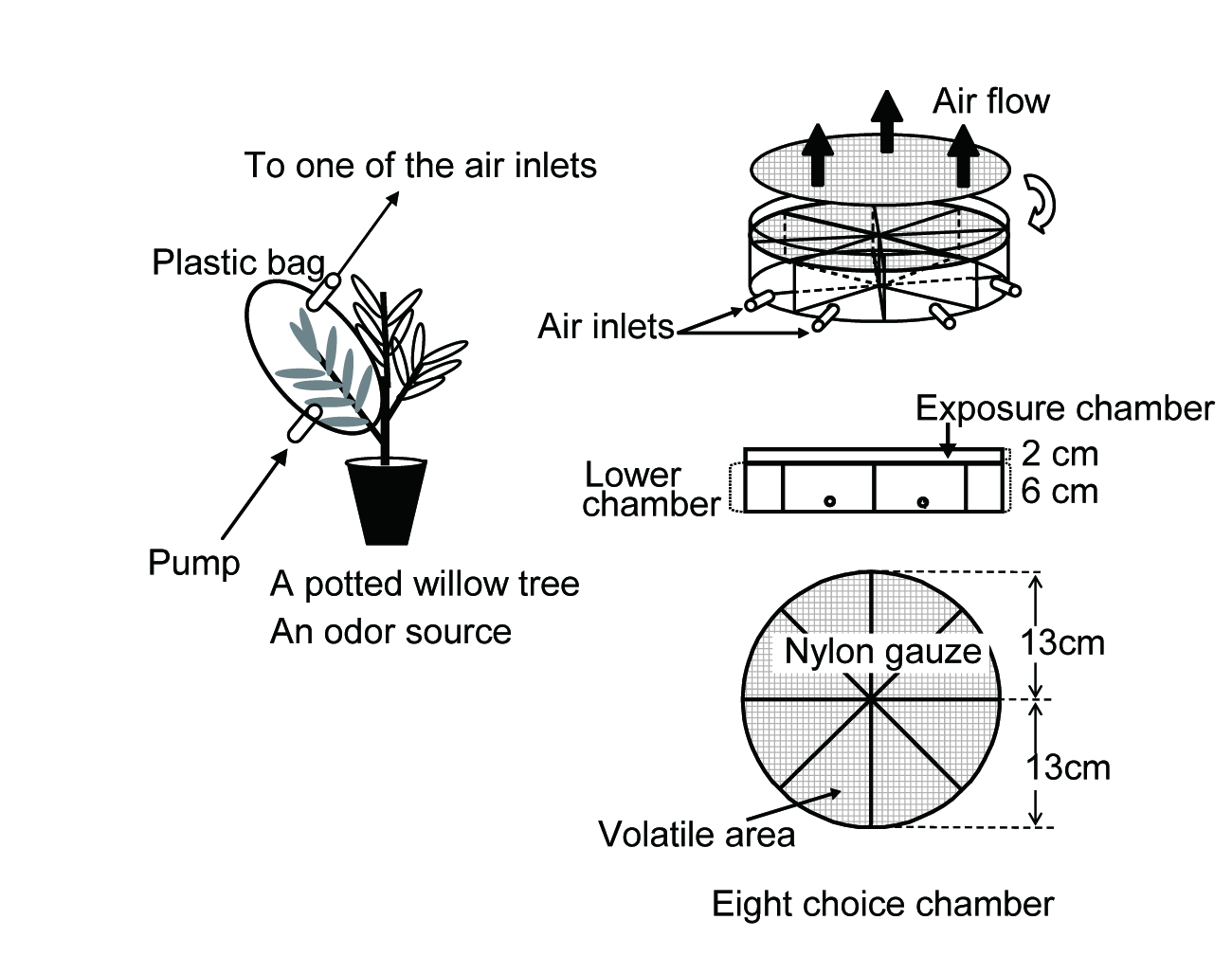

Supplement: Figure S1 — Eight choice chamber. Volatiles from a shoot of a potted willow plant were directed to seven of eight air inlets; the eighth inlet received clean air. The positions of the eight odour sources were changed randomly to remove any positional preferences of the ladybeetles. (TIF) [file pone.0051505.s001.tif]
