# Supplementary material for: An Apparent Trade-Off between Direct and Signal-Based Induced Indirect Defence against Herbivores in Willow Trees
Source: PLoS One. 2012 Dec 12;7(12):e51505. doi: 10.1371/journal.pone.0051505 (PMC3520792; doi:10.1371/journal.pone.0051505)
Supplement: Table S1 — Volatiles detected in the headspace of plants of the seven Salix species infested by P. versicolora larvae. (DOC) [file pone.0051505.s002.doc]

Table S1. Volatiles detected in the headspace of plants of the seven *Salix* species infested by *P. versicolora* larvae

Peak area [mean (s. e.)] (N = 4)

Erio^*^ Chae^*^ Jess^*^ Inte^*^ Miya^*^ Grac^*^ Tria^*, †^ tau (*P*)‡

(*E*)-2-Hexenal 0.69 (0.24) 2.13 (0.77) 0.16 (0.09) 0.66 (0.22) 5.23 (1.72) 0.47 (0.24) 12.30 (3.59) - 0.05 (n.s.)

(*Z*)-3-Hexen-1-ol 2.44 (0.75) 0.68 (0.42) 4.18 (2.35) 5.52 (1.20) 12.68 (4.35) 1.65 (0.85) 7.88 (1.32) - 0.24 (n.s.)

(*Z*)-3-Hexenyl acetate 2.98 (0.65) 1.15 (1.15) 11.31(6.86) 3.92 (1.86) 24.73 (16.41) 1.30 (1.30) 4.55 (0.32) - 0.24 (n.s.)

Benzaldehyde 0.38 (0.20) 9.90 (1.38) 1.66 (0.81) 0.30 (0.16) 10.39 (6.97) 0.73 (0.20) 0.57 (0.10) 0.05 (n.s.)

Salicylaldehyde 4.04 (1.25) 3.98 (0.21) 10.64 (4.17) 2.33 (0.62) 22.24 (10.46) - - - - 0.39 (n.s.)

(*Z*)-β-Ocimene 1.28 (0.43) - - - - - - - - 0.73 (0.73) 0.28 (0.28) - 0.06 (n.s.)

(*E*)-β-Ocimene 15.59 (4.39) 30.73 (6.40) 35.98 (17.34) 4.85 (2.55) 15.02 (5.85) 8.69 (8.69) 10.77 (2.85) 0.14 (n.s.)

*allo*-Ocimene 0.59 (0.23) 0.92 (0.25) 0.72 (0.29) - - - - 0.28 (0.28) - - 0.31 (n.s.)

Linalool 0.20 (0.07) 0.37 (0.37) 1.97 (1.17) - - 1.72 (0.66) 0.66 (0.48) - - - 0.10 (n.s.)

(*E*)-4,8,-Dimethyl-1,3,7 1.01 (0.28) - - 4.62 (2.66) 0.32 (0.32) 3.89 (1.90) 4.07 (0.41) 3.49 (0.89) - 0.23 (n.s.)

-nonatiene

(*E*)-2,6-Dimethyl-1,3,5,7 0.92 (0.20) 1.99 (0.28) 2.40 (1.08) - - - - 0.56 (0.56) - - 0.21 (n.s.)

-octatetraene

(*E*,*E*)-α-Farnesene 0.64 (0.42) 0.16 (0.04) 1.07 (0.98) 0.46 (0.26) 0.34 (0.10) 0.53 (0.30) 0.22 (0.13) 0.05 (n.s.)

(*syn*)- or (*anti*)- 2-Methylbutanal 6.68 (1.92) 21.79 (4.33) 13.07 (4.38) 1.47 (0.85) 10.90 (5.06) 9.39 (5.91) - - 0.24 (n.s.)

oxime

(*syn*)- or (*anti*)- 2-Methylbutanal 3.15 (0.64) 9.03 (1.10) 8.05 (3.63) 0.97 (0.56) 3.99 (1.52) 3.54 (2.26) - - 0.24 (n.s.)

oxime

(*syn*)- or (*anti*)- 3-Methylbutanal 0.62 (0.22) 1.41 (0.83) 2.93 (2.93) - - - - 2.29 (1.77) - - 0.24 (n.s.)

oxime

2-Methylbutanenitrile 3.89 (1.46) 3.88 (1.48) 30.22 (9.27) 1.37 (0.79) 15.90 (6.96) 8.45 (2.65) - - 0.05 (n.s.)

3-Methylbutanenitrile 1.34 (0.70) 0.43 (0.29) 11.44 (4.07) 0.31 (0.18) 5.98 (3.23) 3.44 (0.87) - - 0.05 (n.s.)

Total 46.45(2.98) 88.55(14.75) 140.43 (48.77) 22.47 (1.73) 133.02 (59.33) 46.78 (26.77) 40.06 (1.80) - 0.05 (n.s.)

^*^Erio: *S. eriocarpa*; Chae: *S. chaenomeloides*; Inte: *S. integra*; Miya: *S. miyabeana*; Jess: *S. jessoensis*; Grac: *S. gracilistyla* and Tria: *S. triandra*.

^†^Artificially damaged.

‡Kendall's rank correlation tau (n.s. *P* >0.05)
