# Supplementary material for: An Apparent Trade-Off between Direct and Signal-Based Induced Indirect Defence against Herbivores in Willow Trees
Source: PLoS One. 2012 Dec 12;7(12):e51505. doi: 10.1371/journal.pone.0051505 (PMC3520792; doi:10.1371/journal.pone.0051505)
Supplement: Table S2 — Volatiles detected in the headspace of uninfested plants of the seven Salix species. (DOC) [file pone.0051505.s003.doc]

Table S2. Volatiles detected in the headspace of intact plants of the seven Salix species

Compound name Peak area [mean (s.e.)] (N = 4)

Erio^*^ Chae^*^ Jess^*^ Inte ^*^ Miya^*^ Grac^*^ Tria^*^

(*E*)-2-Hexenal 0.12 (0.12) 1.07 (1.07) - - - - - - - - - -

(*Z*)-3-Hexen-1-ol - - 44.89 (11.45) 3.51 (1.21) 7.59 (3.47) 24.08 (4.87) - - 0.84 (0.56)

(*Z*)-3-Hexenyl 1.16 (0.55) 128.91 (24.32) 48.97 (10.84) 15.13 (9.99) 27.43 (8.09) 3.27 (3.27) 4.08 (1.14)

acetate

Benzaldehyde 0.78 (0.56) 1.38 (0.15) 1.04 (0.43) - - 0.39 (0.24) 0.98 (0.23) 1.15 (0.43)

(*E*)-β-Ocimene 0.07 (0.07) 56.27 (47.76) - - - - - - - - - -

(*E*)-4,8-Dimethyl - - - - 0.02 (0.02) - - 1.81 (0.30) 0.43 (0.43) 1.01 (0.18)

-1,3,7-nonatiene

(*E*,*E*)-α-Farnesene 0.72 (0.72) - - - - - - - - - - - -

Total 2.86 (1.03) 232.5 (12.23) 45.23 (12.54) 22.72 (78.72) 53.71 (12.12) 4.69 (3.64) 7.07 (1.61)

^*^Erio: *S. eriocarpa*; Chae: *S. chaenomeloides*; Inte: *S. integra*; Miya: *S. miyabeana*; Jess: *S. jessoensis*; Grac: *S. gracilistyla* and Tria: *S. triandra*.
