# Supplementary material for: An Apparent Trade-Off between Direct and Signal-Based Induced Indirect Defence against Herbivores in Willow Trees
Source: PLoS One. 2012 Dec 12;7(12):e51505. doi: 10.1371/journal.pone.0051505 (PMC3520792; doi:10.1371/journal.pone.0051505)
Supplement: Table S3 — Scores (coordinates) of volatile compounds. The scores were calculated by weighting the correlation between sample scores (coordinates: each plot in Figure 4a (PCoA)) and vectors of compounds by the variance explained by the first two PCoA axes scores obtained using the Bray-Curtis measurement. (DOC) [file pone.0051505.s004.doc]

**Table S3** Scores (coordinates) of volatile compounds. The scores were calculated by weighting the correlation between sample scores (coordinates: each plot in Fig. 6) and vectors of compounds by the variance explained by the first two PCoA axes scores obtained using the Bray-Curtis measurement.

Dim^*^ 1 Dim^*^ 2

(*E*)-2-Hexenal -0.01 0.08

(*Z*)-3-Hexen-1-ol 0.05 0.60

(*Z*)-3-Hexenyl acetate 0.10 0.50

Benzaldehyde -0.10 -0.13

Salicylaldehyde 0.06 0.27

(*Z*)-β-ocimene -0.02 0.00

(*E*)-β-Ocimene -0.88 -0.01

all-Ocimene -0.02 -0.02

LINALOOLL 0.02 -0.01

(*E*)-4,8,-Dimethyl-1,3,7

-nonatiene 0.23 -0.14

(*E*)-2,6-Dimethyl -0.05 -0.04

-1,3,5,7-octatetraene

(*E*,*E*)-α-Farnesene -0.02 0.02

(*syn*)- or (*anti*)-2

-Methylbutanal oxime -0.20 -0.48

(*syn*)- or (*anti*)-2

-Methylbutanal oxime -0.08 -0.18

(*syn*)- or (*anti*)- 3

-Methylbutanal oxime 0.01 -0.06

2-Methylbutanenitrile 0.74 -0.26

3-Methylbutanenitrile 0.32 -0.10

^*^Dimensions of PCoA (Fig 6)
