# Supplementary material for: An Apparent Trade-Off between Direct and Signal-Based Induced Indirect Defence against Herbivores in Willow Trees
Source: PLoS One. 2012 Dec 12;7(12):e51505. doi: 10.1371/journal.pone.0051505 (PMC3520792; doi:10.1371/journal.pone.0051505)
Supplement: Data S1 — Preferential attraction of ladybeetles to volatiles from uninfested Salix eriocarpa over clean air plus prey. (DOCX) [file pone.0051505.s005.docx]

**Data S1 Preferential attraction of ladybeetles to volatiles from uninfested *Salix eriocarpa* over clean air plus prey**

Figure S2 The response of predatory ladybeetles *Aiolocaria hexaspilota* to volatiles in a Y-tube olfactometer: volatiles from clean air plus larvae of leaf beetles *Plagiodera versicolora* and volatiles from uninfested willow plants *Salix eriocarpa*. Numbers within the bars show the number of individuals that reached the end of the Y-tube.

*A. hexaspilota* adults were significantly more attracted to uninfested *S. eriocarpa* plants (goodness-of-fit test, GT = 5.15, P = 0.16; heterogeneity among samples: GH = 1.20, P = 0.54; pooled effect of treatment: GP = 3.95, P <0.05; Fig. 1a) than to *P. versicolora* larvae only when we focused on pooled data but not when consider the effects of replication.

**Materials and Methods for this data**

*Plants*

In May 2005, a total of 50 1–2-year-old shoots (length 18 cm; no leaves at this time of year) were cut from *Salix eriocarpa* trees growing in the floodplain of the Yasu River in Shiga Prefecture, Japan. They were grown as described in the Methods of the main manuscript.

*Insects*

Adults and egg clutches of *Plagiodera versicolora* and *Aiorocaria hexaspilota* were collected in the floodplain of the Yasu River in Shiga Prefecture, Japan, from April to October 2005. The resulting colony was maintained as reported in Yoneya et al. 2009 (21).

*Olfactometer experiments*

To test the behavioural responses of ladybeetle adults to volatiles, a Y-tube olfactometer was used with each arm connected to a separate glass vessel (2 L) containing a distinct odour source (Sabelis & van de Baan, 1983; Takabayashi & Dicke, 1992). In one vessel, a plant with intact leaves was offered and, in the other, 10 larvae of *P. versicolora* were offered. In this way, we investigated whether ladybeetles responded to intact plant volatiles as a signal for potential prey*.* Air in the olfactometer was cleaned by forcing ambient air through a glass container filled with granular activated charcoal (500 mL) and then through tubes to the arms of the Y-tube (3 L min^−1^ for each arm). Adults of *A. hexaspilota* were introduced individually onto a wire runway that ran through the centre of the tube, parallel to the tube walls. The ladybeetle was observed until it reached the far end of one of the arms. Ladybeetles that did not reach the far end of either arm within 3 min were interpreted as having made ‘no choice’ and were excluded from statistical analyses. The arms containing the plant and insect odours in the olfactometer were switched after every five individuals tested. *Aiolocaria hexaspilota* showed no preference when clean air was offered on both sides. Experiments were replicated three times on different days. Odour source plants and larvae were used once for each replicate, and 10 ladybeetles were used in a replicate. Individual ladybeetles were used only once. The tests were conducted in a climate-controlled room at 25 ± 2°C and 55 ± 5% RH. The distribution of beetles between the two odour sources was analysed using replicated goodness-of-fit tests (Sokal & Rohlf, 1995) under the null hypothesis that ladybeetles had a 1:1 distribution.
